# Interaction between CYP3A4 gene polymorphism and obesity on breast cancer susceptibility in Chinese women

**DOI:** 10.1265/ehpm.25-00022

**Published:** 2025-11-15

**Authors:** Jiamin Zhu, Xiaogang Zhai, Feng Ni, Cheng Tan, Yun Guan, Baixia Yang, Jing Cai

**Affiliations:** 1 Department of Oncology, the Affiliated Jiangyin Hospital of Nantong University, Jiangyin City, Jiangsu Province, 214400, China; 2 Department of Radiotherapy, Nantong Tumor Hospital, Tumor Hospital Affiliated to Nantong University, Nantong City, Jiangsu Province, 226300, China; 3 Department of Radiotherapy, Nantong Rici Hospital Affiliated to Yangzhou University, Nantong City, Jiangsu Province, 226010, China

**Keywords:** Breast cancer, Cytochrome P450, SNP, CYP3A4, Interaction, Obesity, Haplotype

## Abstract

**Background:**

To date, results on relationship between *CYP3A4* gene polymorphism were limited and inconclusive, and no study focused on the influence of *CYP3A4* gene-obesity interaction on breast cancer risk, especially in Chinese women. The purpose of this study was to evaluate the impact of four single nucleotide polymorphisms (SNPs) of CYP3A4 gene, the SNP-SNP and gene-environment interactions on the susceptibility to breast cancer in Chinese women.

**Methods:**

Logistic regression was used to explore the relationship between four SNPs of CYP3A4 gene and the risk of breast cancer. Generalized multifactor dimensionality reduction (GMDR) was used to screen the best SNP-SNP and gene-abdominal obesity interaction combinations among four SNPs and abdominal obesity. Haplotype examination among 4 SNPs was conducted using the SHEsis web-based platform.

**Results:**

Logistic regression analysis showed that carriers of rs2242480- T allele have significantly higher breast cancer risk, than those with rs2242480- CC genotype, adjusted OR (95%CI) was 1.68 (1.23–2.16) and 2.03 (1.53–2.58) for participants with CT genotype and TT genotype under additive model. We did not find any notable interactions between the four SNPs within the CYP3A4 gene. GMDR model found a significant association in a two-locus model involving rs2242480 and obesity, with a *p*-value of 0.018. Stratified analysis found that breast cancer risk was the highest in obese participants with rs2242480- CT or TT genotype, compared to those non-obese participants with rs2242480- CC genotype, OR (95%CI) was 3.02 (1.83–4.25). We found that all haplotype combinations were not correlated with breast cancer risk.

**Conclusions:**

We found that the T allele of rs2242480 within the CYP3A4 gene and interaction between rs2242480 and obesity were associated with an increased risk of breast cancer. However, the results of this study were only applicable to the Han ethnic group and cannot be generalized to other ethnic groups in China, and more SNPs of CYP3A4 gene should been enrolled in the analysis in the future, to verify the results obtained in this study.

## 1. Introduction

Breast cancer stands as the prevalent malignancy affecting women globally [1], and accounts for about 25% of the total number of newly diagnosed cancer cases in women worldwide [2]. Since the early 20th century, breast cancer incidence and mortality rates in China have steadily increased, making breast cancer an important public health problem in the country [3, 4]. Studies [5, 6] have shown that the pathogenesis of cancer is not very clear and involves numerous risk factors, including genetic factors, environmental factors, hormone levels, lifestyle, etc.

Cytochrome P450 (CYPs) is a complex group of enzymes that play a crucial role in the metabolism of compounds such as steroids, estrogen, and progesterone [6, 7]. CYP cyclooxygenase enzymatically converts arachidonic acid into epoxyeicosatrienoic acids, crucial molecules implicated in the progression of diverse cancer types [8]. The CYP3A4 gene is located in the Cytochrome P450 gene cluster on chromosome 7q21.1 and plays an important role in the pathogenesis of breast cancer [9–11]. However, to date, results on relationship between *CYP3A4* gene polymorphism were limited and inconclusive. The CYP3A4 polymorphism is significantly associated with early onset of puberty, which increases the risk of breast cancer [12]. The CYP3A4 gene is engaged in the inactivation and biotransformation of one-third of the drugs used clinically [13]. In addition, it had been found that CYP3A4 genes could inactivate the anti-tumor drug docetaxel and exacerbate its side effects. Therefore, exploring the impact of CYP3A4 gene polymorphism on the risk of breast cancer was of great clinical significance [14].

Recently, epidemiological studies [15, 16] suggested a correlation between overweight and obesity and an increased risk of breast cancer. Additionally, it was proven that the development of breast cancer is the result of a complex interaction between genes and the environment [17, 18]. Recent study [19] suggested that obesity-induced epigenetic variations increased the risk of breast cancer, and a study [20] also reported that the interaction between gene polymorphism and obesity could influence breast cancer risk. However, no study focused on the influence of *CYP3A4* gene-obesity interaction on breast cancer risk yet, especially in Chinese women. Considering the aforementioned reasons, including limited previous research, inconsistent research results, and the lack of interaction analysis between *CYP3A4* gene and obesity, we therefore performed this study to evaluate the relationship between four single nucleotide polymorphisms (SNPs) of *CYP3A4* gene and susceptibility to breast cancer, and the impact of SNP-SNP and gene-obesity interactions on breast cancer susceptibility in Chinese women.

## 2. Materials and methods

### 2.1 Subjects

A total of 1176 participants including 586 breast cancer patients and 590 normal controls were enrolled. We selected breast cancer patients from those patients. We randomly selected 590 cases from those pathologically confirmed breast cancer patients who were hospitalized in the Affiliated Jiangyin Hospital of Nantong University and the Nantong Tumor Hospital between April 2019 and March 2023. Two patients with others primary tumors and two patients who has missing information were excluded from the case group. Finally, 586 eligible breast cancer cases were included in this study. The normal controls were randomly selected from residents participating in physical health examinations and nearly 1:1 matched to cases based on age (±2 years), those participants with any type of cancer were excluded from the study. All participants were Chinese Han women and were unrelated each other. We collected demographic information, clinical profiles, lifestyle facts, and familial breast cancer histories from each participant. Measurements of body weight, height, and waist circumference were taken, to facilitate the computation of each participant’s body mass index (BMI). Every participant provided their written agreement to partake in the study. Additionally, sample size was calculated using PASS 15.0.5 software [21] (power ≥ 0.95, *α* < 0.05, OR = 2.0, P_2_ = 0.2), the results of which indicated that the sample size of this study was more than the minimum sample size.

### 2.2 Genomic DNA extraction and genotyping

Blood samples were obtained from the peripheral veins of all participants and were stored in EDTA-coated tubes at −80 °C until further analysis. We extracted genomic DNA from the study participants’ whole blood, treated with EDTA, by utilizing the DNA Blood Mini Kit from Qiagen (Hilden, Germany), and we adhered strictly to the guidelines provided by the kit’s producer. The genotypes of four SNPs within *CYP3A4* gene were genotyped by using PCR–RFLP method. The total reaction volume of PCR reaction was 20 µL, which contained: 50 ng genomic DNA, 1x PCR buffer, 2 mM MgCl2, 200 µm each dNTP, 0.1 µm each primer, information of which was shown in Table 1. PCR conditions involved: initial denaturation at 98 °C for 5 min, followed by denaturation at 95 °C for 30 s, annealing at 60 °C for 45 s, extension at 72 °C for 45 s, and final extension at 72 °C for 5 min. The PCR conditions were as follows: an initial denaturation at 98 °C for 5 min, followed by denaturation at 95 °C for 30 s, annealing at 60 °C for 45 s, extension at 72 °C for 45 s, and a final extension at 72 °C for 5 min. We evaluated genotyping precision through two methods. First, we inspected the efficacy of marker and sample genotyping, along with the results from both positive and negative controls. Second, we randomly selected approximately 10% of the samples for re-genotyping, and the reproducibility rate reached 100%.

**Table 1:** Description for 4 SNPs of *CYP3A4* gene and primers used for genotyping

**ID**	**Chromosome**	**Functional Consequence**	**Primer sequences**
rs2242480 C>T	7:99763843 (GRCh38) 7:99361466 (GRCh37)	Intron variant	Forward: 5′-CTGGCTATGAAACCACGAGC-3′ Reverse: 5′-TCTGCCAGTAGCAACCATTTG-3′
rs4646437 G>A	7:99767460 (GRCh38) 7:99365083 (GRCh37)	Intron variant	Forward 5′-CAAAGAATCCCAATTTTGGCAGAG-3′ Reverse 5′-TCAGTCCCTGGGGTGAGAG-3′
rs3735451 C>T	7:99758352 (GRCh38) 7:99355975 (GRCh37)	Intron variant	Forward: 5′-ACGTTGGATGCAAAGTGAGTGAGACACTCC-3′ Reverse: 5′-ACGTTGGATGTACTGCATTTTTTTTGCCC-3′
rs2740574 C>T	7:99784473 (GRCh38) 7:99382096 (GRCh37)	2KB upstream variant, upstream transcript variant	Forward: 5′-CACCACTCACTGACCTCCTT-3′ Reverse: 5′-GCACACTCCAGGCATAGGTA-3′

### 2.3 Statistical analysis

In this study, we employed SPSS 22.0 software package (SPSS Inc, Chicago) to complete most statistical analyses. Demographic and clinic data of breast cancer patients and controls were compared using Student’s *t* test and χ^2^ test between two groups. Calculation for frequencies genotype and allele and Hardy-Weinberg equilibrium (HWE) test were performed using SNPstats (http://bioinfo.iconcologia.net/SNPstats). We conducted logistic regression to explore the relationship between four CYP3A4 gene SNPs and the risk of breast cancer, adjusting for age, age at menarche, menopausal status, breast feeding status, number of children, smoking, alcohol drinking and BMI. Generalized multifactor dimensionality reduction (GMDR) [22] was used to screen the best SNP-SNP and gene-abdominal obesity interaction combinations among four SNPs and abdominal obesity. We conducted haplotype examination of SNPs using the SHEsis web-based platform (http://shesisplus.bio-x.cn/SHEsis.html). We considered the *p*-values less than 0.05 to be statistically significant. All the *p*-values were calculated using a two-tailed test.

## 3. Results

In this study, a total of 1176 participants were enrolled, including 586 breast cancer patients and 590 normal controls. The study participants consisted of 470 patients with more than 40 years and 449 controls with more than 40 years. The mean age of all participants was 56.9 ± 11.8 years. Table 2 displayed the traits of participants, categorizing them into groups of breast cancer cases and controls. The distributions for age (≥40 years), menopausal status, breast feeding status, smokers, alcohol consumption and the average age at menarche in breast patients showed no significant disparity when comparing the control subjects. The distribution of cancer family history, number of children and means of BMI and WC have significant differences in breast cases, when compared to normal controls. The means of BMI and WC were higher in breast cancer patients than controls.

**Table 2:** General characteristics of 1176 participants grouped into case and control group

**Variables**	**Breast cancer patients** **(n = 586)**	**Normal controls** **(n = 590)**	***p*-values**
Age (years), N (%)			0.089
<40 years	116 (19.8)	141 (23.9)	
≥40 years	470 (80.2)	449 (76.1)	
Age at menarche (years) (Mean ± SD)	12.6 ± 5.3	13.2 ± 5.9	0.067
Menopausal status, N (%)			0.683
Premenopausal	290 (49.5)	299 (50.7)	
Postmenopausal	296 (50.5)	291 (49.3)	
Breast feeding status, N (%)			0.396
Breast feeding	484 (82.6)	476 (80.7)	
Non-breast feeding	102 (17.4)	114 (19.3)	
Smokers, N (%)	50 (8.5)	44 (7.5)	0.497
Alcohol consumption, N (%)	65 (11.1)	62 (10.5)	0.747
WC (cm) (Mean ± SD)	85.3 ± 14.1	83.5 ± 13.6	0.026
BMI (kg/m^2^) (Mean ± SD)	25.4 ± 10.2	23.8 ± 10.5	0.008
Number of children (≥3), N (%)	117 (20.0)	151 (25.8)	0.021
Cancer family history, N (%)	215 (36.7)	133 (22.5)	<0.001

Table 3 showed the frequencies of the four SNPs genotypes and alleles within CYP3A4 gene under three genetic models (additive, dominant and recessive models). We found that breast cancer patients had higher frequencies for rs2242480- CT/TT genotypes and rs2242480- T allele of CYP3A4 gene than controls. Logistic regression analysis showed that carriers of rs2242480- T allele had significantly higher breast cancer risk, than those with rs2242480- CC genotype, adjusted OR (95%CI) was 1.68 (1.23–2.16) and 2.03 (1.53–2.58) for participants with CT genotype and TT genotype under additive genetic model. We also found a statistical association between rs2242480 and breast cancer in dominant model, adjusted OR (95%CI) was 1.77 (1.33–2.24), but no significant association between rs2242480 and breast cancer in recessive model, adjusted OR (95%CI) was 1.17 (0.71–1.68). However, we also found no significant association of rs4646437, rs3735451 and rs2740574 with breast cancer risk after covariates adjustment in additive, dominant and allele models.

**Table 3:** Genetic association analysis between 4 SNPs within CYP3A4 gene and breast cancer risk

**SNP**	**Genotypes and Alleles**	**Frequencies N (%)**		OR (95%CI)*	***P*-values for HWE ** **test in controls**

		**Cases** **(n = 586)**	**Controls** **(n = 590)**		
rs2242480, C>T					0.680
	Additive				
	CC	297 (50.7)	380 (64.4)	1.00	
	CT	233 (39.8)	185 (31.4)	1.68 (1.23–2.16)	
	TT	56 (9.6)	25 (4.2)	2.03 (1.53–2.58)	
	Dominant				
	CC	297 (50.7)	380 (64.4)	1.00	
	CT+TT	289 (49.3)	210 (35.6)	1.77 (1.33–2.24)	
	Recessive				
	CC+CT	530 (90.4)	565 (95.8)	1.00	
	TT	56 (9.6)	25 (4.2)	1.17 (0.71–1.68)	
	Allele, T (%)	345 (29.4)	235 (19.9)		
rs4646437, G>A					0.137
	Additive				
	GG	323 (55.1)	355 (60.2)	1.00	
	GA	213 (36.3)	197 (33.4)	1.21 (0.80–1.67)	
	AA	50 (8.5)	38 (6.4)	1.43 (0.71–2.18)	
	Dominant				
	GG	323 (55.1)	355 (60.2)	1.00	
	GA+AA	263 (44.9)	235 (39.8)	1.28 (0.78–1.80)	
	Recessive				
	GG+GA	536 (91.5)	552 (93.6)	1.00	
	AA	50 (8.5)	38 (6.4)	1.19 (0.72–1.69)	
	Allele, A (%)	313 (26.7)	273 (23.1)		
rs3735451, C>T					0.176
	Additive				
	CC	324 (55.3)	362 (61.4)	1.00	
	CT	215 (36.7)	193 (32.7)	1.18 (0.71–1.69)	
	TT	47 (8.0)	35 (5.9)	1.30 (0.68–1.93)	
	Dominant				
	CC	324 (55.3)	362 (61.4)	1.00	
	CT+TT	262 (44.7)	228 (38.6)	1.21 (0.70–1.74)	
	Recessive				
	CC+CT	539 (92.0)	555 (94.1)	1.00	
	TT	47 (8.0)	35 (5.9)	1.12 (0.65–1.66)	
	Allele, T (%)	309 (26.4)	263 (22.3)		
rs2740574, C>T					0.212
	Additive				
	CC	328 (56.0)	357 (60.5)	1.00	
	CT	207 (35.3)	197 (33.4)	1.06 (0.64–1.53)	
	TT	51 (8.7)	36 (6.1)	1.28 (0.57–2.02)	
	Dominant				
	CC	328 (56.0)	357 (60.5)	1.00	
	CT+TT	258 (44.0)	233 (39.5)	1.10 (0.62–1.61)	
	Recessive				
	CC+CT	535 (91.3)	554 (93.9)	1.00	
	TT	51 (8.7)	36 (6.1)	1.15 (0.61–1.84)	
	Allele, T (%)	309 (26.4)	269 (22.8)		

*Adjusted for age, age at menarche, menopausal status, breast feeding status, number of children, smoking, alcohol drinking and BMI.

The SNP-SNP and gene-abdominal obesity interactions were evaluated by using GMDR model in this study. Table 4 presented a summary of the outcomes from the GMDR evaluation model, focusing on SNP-SNP and gene-obesity interactions. The GMDR method calculated three model parameters, including cross validation consistency (CVC), testing balance accuracy, training balanced accuracy and *P*-value. The cross-validation consistency of the model refers to the number of times the same GMDR model is identified across all possible training sets in 10-fold cross-validation training. The model with the highest prediction accuracy and maximum cross validation consistency score is defined as the best model. Our analysis did not find any notable interactions between the four SNPs within the CYP3A4 gene. However, we found a significant combination involving rs2242480 and obesity in a two-locus model (*p*-value = 0.018, CVC = 10/10, the testing balance accuracy = 0.632 and training balanced accuracy = 0.668).

**Table 4:** Best models to predict breast cancer by generalized multifactor dimensionality reduction (GMDR) models

**Locus no.**	**Best combination**	**Cross-validation consistency**	**Training balanced accuracy**	**Testing balanced accuracy**	*p*-values*
SNP-SNP interactions*					
2	rs2242480 and rs4646437	8/10	0.613	0.496	0.245
3	rs2242480, rs4646437 and rs3735451	7/10	0.618	0.515	0.614
4	rs2242480, rs4646437, rs3735451 and rs2740574	6/10	0.582	0.513	0.624
Gene-obesity interactions**					
2	rs2242480 and obesity	10/10	0.668	0.632	0.018
3	rs2242480, rs4646437 and obesity	9/10	0.613	0.513	0.213
4	rs2242480, rs4646437, rs3735451 and obesity	8/10	0.596	0.521	0.377
5	rs2242480, rs4646437, rs3735451, rs2740574 and obesity	7/10	0.586	0.524	0.532

*Adjusted for age, age at menarche, menopausal status, breast feeding status, number of children, smoking, alcohol drinking and BMI.

**Adjusted for age, age at menarche, menopausal status, breast feeding status, number of children, smoking and alcohol drinking.

**Fig. 1:**
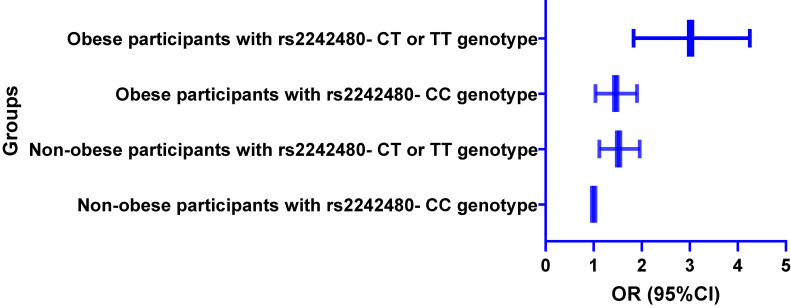
Stratified logistic analysis on interaction between rs2242480 and obesity on breast cancer risk *Adjusted for age, age at menarche, menopausal status, smoking, breast feeding status, number of children, smoking and alcohol drinking.

We also grouped participants into 4 groups according to rs2242480 genotypes (dominant model) and obesity, including non-obese participants with rs2242480- CC genotype, non-obese participants with rs2242480- CT or TT genotype, obese participants with rs2242480- CC genotype, obese participants with rs2242480- CT or TT genotype. We performed stratified analysis by logistic regression, and we found that breast cancer risk was the highest in obese participants with rs2242480- CT or TT genotype, compared to those non-obese participants with rs2242480- CC genotype, OR (95%CI) was 3.02 (1.83–4.25) (Fig. 1).

Examining haplotypes may offer a superior method for risk prediction. So, we also performed pairwise LD analysis among the 4 SNPs, we found that heavy LD existed between rs2242480 and rs4646437 (D′ value was 0.862). Then, we employed SHEsis online software for haplotype analysis between rs2242480 and rs4646437. The haplotype composed of C and G has the most frequencies in both groups (54.37% for patients and 58.42% for controls). Conversely, the T and A combination has the least frequencies (3.46% in patients and 2.53% in controls). However, we found no haplotype was correlated with the breast cancer risk (Table 5). The negative results indicated that the haplotype of rs2242480 and rs4646437 had no significant impact on the risk of breast cancer.

**Table 5:** Association of *CYP3A4* gene haplotypes with breast cancer risk

**Haplotypes (rs2242480 and rs4646437)**	**Frequencies**		**OR (95%CI)**	*p*-values*

	**Case group**	**Control group**		
C- G	0.5437	0.5842	1.00	-
C- A	0.2402	0.2216	1.28 (0.71–1.90)	0.472
T- G	0.1815	0.1689	1.16 (0.53–1.83)	0.581
T- A	0.0346	0.0253	1.63 (0.51–2.78)	0.723

*Adjusted for age, age at menarche, menopausal status, breast feeding status, number of children, smoking, alcohol drinking and BMI.

## 4. Discussion

In this study, we found that rs2242480 within CYP3A4 gene was significantly associated with increased breast cancer risk. However, we found no significant association of rs4646437, rs3735451 and rs2740574 with breast cancer risk after covariates adjustment in additive, dominant and allele genetic models. Our study was not the first case-control study to verify the impact of CYP3A4 gene SNP on the risk of breast cancer. However, considered the inconsistent results of previous studies, especially in the Chinese population, we believed that our results were necessary and meaningful for understanding the mechanism of breast cancer, especially for the susceptibility caused by CYP3A4 genetic polymorphism. Regarding the rs2242480 polymorphism of the CYP3A4 gene, the T allele frequency in this study was 24.66%, which is similar with other reports in Han and Asian populations [23], but significantly different from the T allele frequencies in Caucasian (7.34%) and African (85.71%) populations. The rs2242480 polymorphism within the CYP3A4 gene had shown an association with certain cancers, such as those affecting the prostate, breast, and ovaries [24–26]. Previously, several studies [11, 27, 28] had reported that the rs2242480 polymorphism of CYP3A4 gene had a significant impact on the risk of breast cancer, but these studies yielded negative results. Parada et al [11] performed a case-control study and showed that the CYP3A4 gene rs2242480 played a significant role in the development of breast cancer. But AL-Eitan et al [10] performed a case-control study, which enrolled 231 healthy controls and 242 breast cancer patients, suggested that CYP3A4 gene SNPs was not associated with breast cancer risk in the Jordanian population. The sample size of this study was smaller than ours, so limited number of patients could influence the results stability. A case-control study performed in Chinese Han population by Liu et al [29] provided evidence for impact of *CYP3A4*- rs2242480 polymorphism on the development of breast cancer. The inconsistent results concluded by previous studies were due to including different populations and having a relatively small sample size. And in our study, more breast cancer patients and controls were enrolled, and the method of approaching 1:1 matching was used to determine the control population, so the results would be more stable and reliable. In terms of CYP3A4 gene rs2740574, Veiga et al [30] indicated that CYP3A4 gene rs2740574 (CYP3A4*1B) did not play an important role in development of breast cancer in the Portuguese population. And in this study, we also found no correlation between CYP3A4 gene rs2740574 and breast cancer risk.

Variations in the activity levels of enzymes such as CYP3A4 could modify the amounts of hormones like estradiol and progesterone in the bloodstream. These changes might also affect the generation of reactive compounds and the clearance of steroid hormone byproducts, consequently impacting breast cancer risk [31, 32]. Additionally, studies had noted an increased expression of CYP3A4 in breast cancer cells, which was implicated in processes like cell growth, blood vessel formation, and cellular movement, partly through the production of substances like 11,12- EET [33]. Considering the pivotal role of CYP3A4 for processing natural hormones and its involvement in breast cancer development, specific genetic variations in the CYP3A4 gene- particularly those that might change the enzyme’s performance or response compared to typical alleles- could potentially influence the likelihood of developing breast cancer [34].

The pathogenesis of breast cancer was complex and not well known, and its risk was affected by genetic factors, environmental factors and their interactions. Hence, it was necessary to investigate the impact of SNP-SNP and gene-environment interactions on breast cancer risk. Wu et al [35] suggested a significant SNP-SNP interaction among acylphosphatase 2 gene SNPs in Chinese Han females. However, we found no interaction among CYP3A4 gene SNPs. Previous studies [36, 37] reported a significant interaction between obesity and other genes in different populations. They found that obesity had a significant role in modification of epigenetic and could increase breast cancer risk. In addition, Nam et al [20] suggested a significant interaction between adiponectin-related SNPs and obesity on breast cancer risk among African American Women. In this study, we first investigated the impact of CYP3A4 gene-obesity interaction. We found a significant interaction involving rs2242480 and obesity, obese participants with rs2242480- CT or TT genotype had the highest breast cancer risk, compared to non-obese participants with rs2242480- CC genotype. In this study, we also performed haplotype analysis to verify the influence of CYP3A4 gene four SNPs on breast cancer risk. Although heavy LD existed between rs2242480 and rs4646437, we did not find any haplotype combination associated with breast risk. Breast cancer is a complex genetic disease; the haplotype of a single gene had a weak impact on breast cancer. In addition, relative smaller sample size may lead to negative results.

Several limitations existed in the current study. First, the sample’s homogeneity, all participants in this study were from the Han Chinese women, therefore the results of this study were only applicable to the Han Chinese women and could not be generalized to other ethnic groups in China; Second, just four SNPs were enrolled in this study, and more SNPs of CYP3A4 gene should been selected in the future to verify the potential SNP-SNP synergy effect. Thirdly, the lack of longitudinal data could lead to the instability of the results, so the results obtained in our study also need to be verified by prospective study. In the future, we suggest that further studies should be performed to explore other SNPs in CYP3A4 or investigating other gene-environment interactions contributing to breast cancer risk.

In conclusion, we found that the T allele of rs2242480 within CYP3A4 gene, their interaction between rs2242480 and obesity were all associated with increased breast cancer risk. Although, T allele of rs2242480 within CYP3A4 could influence breast cancer risk, this effect could be influenced by some environmental factors, such as obesity. It was a known entity that the genetic characteristics of the Asian society were different from the western society, therefore drug toxicity and drug efficacy may be different. So, this different genetic structure could cause cancer susceptibility in addition to other known cancer related genes. Obesity was an important risk factor for breast cancer in female, and controlling weight could reduce the risk of breast cancer. Therefore, weight management was recommended as a preventive strategy for breast cancer. Especially for obese individuals carrying the rs2242480 mutation site, the findings of this study had important guiding significance for early screening in the Han population in China. In the future, the relationship between *CYP3A4* gene SNPs and its interaction with obesity should be verified in different populations, and more environmental factors should be included in the interaction analysis.

## Abbreviations

CYPs: Cytochrome P450
BMI: Body mass index
SNPs: single nucleotide polymorphisms
GMDR: Generalized multifactor dimensionality reduction
SD: standard deviation
HWE: Hardy-Weinberg equilibrium
WC: Waist circumference

## Declarations

### Data availability statement

The data and figures generated and analyzed during this study are available from the corresponding authors upon reasonable request.
